# Immediate Postoperative Pain and Recovery Time after Pulpotomy Performed under General Anaesthesia in Young Children

**DOI:** 10.1155/2017/9781501

**Published:** 2017-06-08

**Authors:** Sultan Keles, Ozlem Kocaturk

**Affiliations:** ^1^Department of Pediatric Dentistry, Faculty of Dentistry, Adnan Menderes University, Aydın, Turkey; ^2^Department of Oral and Maxillofacial Surgery, Faculty of Dentistry, Adnan Menderes University, Aydın, Turkey

## Abstract

**Background:**

The aim of this retrospective study was to compare immediate postoperative pain scores and need for rescue analgesia in children who underwent pulpotomies and restorative treatment and those who underwent restorative treatment only, all under general anaesthesia.

**Methods:**

Ninety patients aged between 3 and 7 years who underwent full mouth dental rehabilitation under general anaesthesia were enrolled in the study and reviewed. The experimental group included patients who were treated with at least one pulpotomy, and the control group was treated with dental fillings only. The Wong-Baker FACES scale was used to evaluate self-reported pain and need for rescue analgesia. The data were analysed using the Kruskal-Wallis test, two sample *t*-tests, chi-square tests, and Pearson's correlation analysis.

**Results:**

Ninety percent of the children experienced postoperative pain in varying degrees of severity. Immediate postoperative pain scores in experimental group were found to be significantly higher than in control group (*x*^2^ = 24.82, *p* < 0.01). In the experimental group, 48% of the children needed rescue analgesia, compared with only 13% of the children in the control group (*x*^2^ = 13.27, *p* < 0.05).

**Conclusion:**

Children who underwent pulpotomy treatment had higher postoperative pain scores and greater need for rescue analgesia than control group who underwent only dental fillings.

## 1. Introduction

Dental caries remains a global public health problem. Young children who are frequently exposed to sugary liquids, poor oral hygiene, breast feeding, fruit juices, and other sweet liquids for long periods are particularly at risk of suffering from extensive caries. Children with untreated caries may experience infection, pain, disturbed sleep, speech and communication problems, and inability to eat leading to weight loss [1].

Treatment of young children frequently requires a multidisciplinary treatment plan that includes general anaesthesia (GA) because they may be unable to cooperate in a dental clinic setting. Despite the fact that GA is an accepted behavior management technique according to the American Academy of Pediatric Dentistry, this procedure has some morbidity or mortality risks. Postoperative pain is the most common morbidity after GA for dental rehabilitation in children [2].

Tooth extraction and pulp therapies of primary teeth (pulpotomy and pulpectomy) have been designated as painful dental procedures (PDPs) in previous studies, and postoperative pain may occur after these procedures [3]. Pulpotomy is a vital pulp therapy approach and can be defined as the surgical removal of the coronal pulp of the vital and reversibly inflamed tooth. Main goals of this treatment are preserving the radicular pulp in a healthy state, rendering the radicular pulp inert, and encouraging tissue regeneration [4]. During the presence of irreversibly inflamed or necrotic radicular pulp tissue, pulpectomy can be considered as a treatment option [4]. Ashkenazi et al. [5] evaluated postoperative pain after various dental procedures and reported that endodontic procedures induced a significantly higher incidence of postoperative pain compared to restorations. Staman et al. [6] determined that children who received pulpotomy and stainless steel crown had higher postoperative pain than other dental procedures.

Effective postoperative pain management is important in reducing the recovery time, improving patient outcomes, and decreasing the length of hospital stays [7]. There have been many attempts to decrease postoperative pain in children after painful dental procedures under GA, but there is no consensus on how to best manage postoperative pain. Some researchers have reported that local anaesthetic injections lessened postoperative pain after tooth extractions under GA in young children [8]. Others found that local anaesthetic usage had no effect on postoperative pain management [9]. During recent years, most investigators have used IV analgesic agents to decrease postoperative pain [10]. Determining the pain scores of children during the postoperative period is a significant step in defining analgesic requirements. Most studies have focused on evaluating postoperative pain after tooth extraction performed under GA [11, 12]. Few studies have evaluated postoperative pain after endodontic procedures under general anesthesia such as pulpotomy, a technique used extensively in pediatric dentistry [9, 10].

For this reason, this study was undertaken to compare the postoperative pain scores of children who underwent primary molar pulpotomy that is a vital endodontic treatment under GA with those of children in the control group who underwent only regular restorative treatment under GA and to evaluate the need for rescue analgesia during the postoperative period. An additional purpose of this study was to investigate the effects on the recovery time of the two treatment types (fillings and pulpotomies) performed under GA.

## 2. Methods

This retrospective cohort study was conducted on children who underwent full mouth dental rehabilitation under general anaesthesia between July 2015 and January 2016. The study was approved (2016/764) by the Research and Ethics Committee of the Adnan Menderes University and registered (Protocol Registration Receipt NCT03142672) at http://www.clinicaltrials.gov.

### 2.1. Study Sample

All the patients' records were reviewed to determine their age at the time of the dental treatment; sex; number of decayed, missing, and filled teeth (dmft) according to World Health Organization (WHO) criteria [14]; type of dental procedure completed; recovery time (RT); American Society of Anesthesiologists (ASA) classification; postoperative Wong-Baker FACES scores; rescue analgesia need [RAN (present or absent)]; and duration of the dental operation (DDO). Data from 169 patients who underwent full mouth dental rehabilitation under GA because of their lack of chairside cooperation were enrolled in the study; those with ASA class 1 status who were aged 3 to 7 years were reviewed.

Patients with at least 1 primary molar pulpotomy and tooth filling were included in the experimental group (*n* = 45), and patients treated with dental fillings only were included in the control group (*n* = 45); a total of 90 patients were eligible for the study.

Children with special needs and those with any systematic problem (cardiac disease, diabetes, or intellectual disability) and had discomfort except postoperative dental pain were excluded from the study. Patients who received pulpectomy and/or extraction were excluded from the study. All patients were elective cases; those preoperative pain scores were “0.”

### 2.2. General Anaesthesia Procedure

All children were examined and verified as fit for GA by a pediatrician on the day before the anaesthesia procedure. On the morning of the treatment, the patients were not allowed to eat or drink for at least six hours before the GA commenced. No premedication was given. All patients were intubated by the same anaesthetist. Induction was carried out via a facemask with 8% sevoflurane in 100% oxygen.

Following loss of consciousness, an intravenous line was established through which propofol 1% (Propofol-Lipuro®, B. Braun Melsungen AG, Germany) 2 mg/kg, fentanyl (Talinat®, Vem, İstanbul, Turkey) 1 *μ*g/kg, rocuronium (Myocron®, Vem) 0.5 mg/kg were given via nasotracheal intubation. Heart rate, respiratory rate, noninvasive blood pressure, oxygen saturation, and end-tidal carbon dioxide were recorded.

Anaesthesia was maintained with sevoflurane 2% in a mixture of 50% oxygen and nitrous oxide. All the children received tenoxicam (Tilcotil®, Deva, Istanbul, Turkey) 0.4 mg/kg for analgesia 15 min before the end of the surgery. Patients were extubated and transferred to the postanesthesia care unit (PACU).

After transfer to PACU, patients were monitored by a registered nurse. The nurse evaluated the patient at 5-minute intervals using the Aldrete scale.  Table 1 presents the components of the Aldrete score. Recovery time was calculated as the time until the Aldrete score reached 9 or more after coming to PACU. All pertinent surgical times and recovery times were recorded.

**Figure 1:**
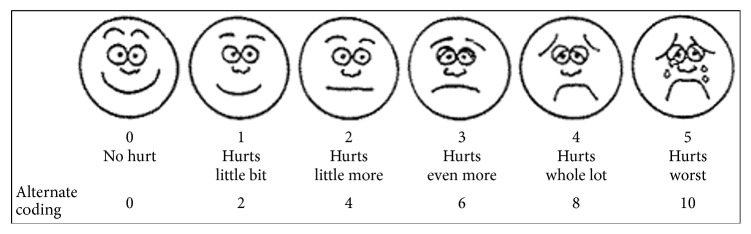
Wong-Baker FACES scale [13].

Patient pain was self-assessed using the Wong-Baker FACES scale immediately, after one hour, and after two hours postoperatively. This scale, which is validated for children aged 3 to 7 years, includes six cartoon faces with varying expressions ranging from very happy to very sad [13] (Figure 1).

When the Wong-Baker FACES score was 4 points or greater during the postoperative period, pain intensity was assessed as moderate-severe, and this was recorded on the patient sheet as a rescue analgesia need [13]. Patients with a score of ≥ 4 points were medicated with IV fentanyl 0.5 *μ*g/kg dose.

### 2.3. Dental Treatment Procedure

All dental treatment was performed by the same paediatric dentist (SK). All restorative and pulpal treatments were completed in a single session under GA. Local analgesia was not used prior to pulpotomies and fillings. After caries removal, all of the primary teeth (primary molar, primary canine, and primary successor) were restored with compomer resin (Dyract®, Dentsply, Konstanz, Germany) according to the manufacturer's recommendations. The pulps of teeth (only primary molars) with extensive caries were removed and bleeding was arrested with gentle pressure from a sterile cotton pledget moistened with saline. Ferric sulfate pulpotomies were performed and primary teeth were restored with compomer resin according to the manufacturer's recommendations. The occlusion was checked in all patients after compomer resin restoration and final finishing and polishing of the restoration was performed using soflex disks (3M-ESPE Dental Products, St. Paul, MN, USA). The duration of the dental operations was recorded in the patient's file.

### 2.4. Statistical Analysis

All numerical and categorical data saved into a database and the reports created in Microsoft Excel format were exported into Statistical Package for Social Science program for Windows (SPSS 17.0, SPSS Inc., Chicago, USA) for statistical analysis. All data were subjected to the Kolmogorov-Smirnov test for normality. For data which were not normally distributed, the Kruskal-Wallis test for independent groups was used for statistical analysis. Pearson's correlation analysis was used to detect the relationship between pain scores and the number of dental procedures performed for each group (control and experimental). Two sample *t*-tests were used to compare normal continuous variables in independent groups, and chi-square tests were used to compare numerical and categorical variables between the two groups. A 5% type-1 error level was used to infer statistical significance.

## 3. Results

The mean age of the participants was 4.6 ± 1.7; 38 were female and 52 were male. No significant differences were found between the two groups for the age and gender parameters (*p* > 0.05). The average dmft of the experimental group was 8.3 ± 3.2 and the average dmft of the control group was 6.6 ± 3.1. In the experimental group, the average number of pulpotomies was 2.6 ± 1.4 and the number of fillings was 5.7 ± 3.3, while in the control group the average number of fillings was 6.6 ± 3.1.

**Figure 2:**
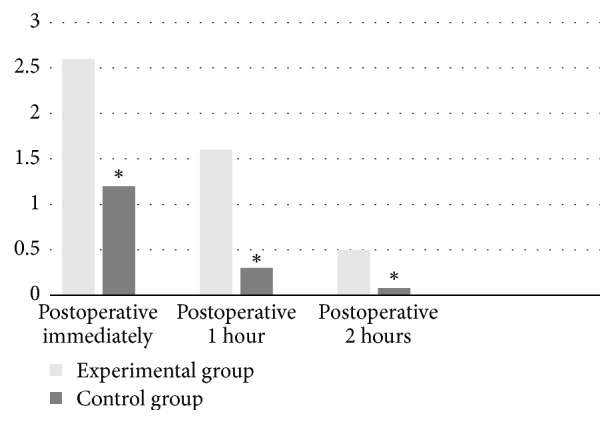
Comparison of postoperative pain scores of groups, *∗* = *p* < 0.05.

Overall 90% of all patients experienced varying degrees of postoperative pain. Immediate postoperative pain scores in the experimental group were significantly higher than those in the control group (*x*^2^ = 24.82, *p* < 0.01). Statistically significant differences were determined between experimental and control groups in terms of immediate, 1-hour, and two-hour postoperative pain scores (*p* < 0.01). The immediate, 1-hour, and 2-hour postoperative average pain scores of the experimental and control groups are shown in Table 2.  Figure 2 shows the comparison of the postoperative pain scores of the two groups.

In the intragroup comparison in the experimental group, no significant relationship was found between the number of pulpotomies and the postoperative pain score (*r* = 0.59, *p* > 0.05). Similarly, in the intragroup comparison in the control group, no significant relationship was observed between the number of fillings and the postoperative pain score (*r* = 0.79, *p* > 0.05).

**Figure 3:**
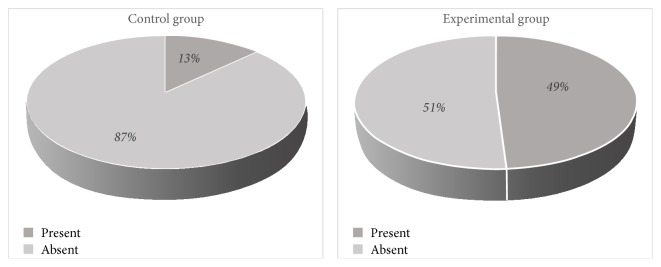
Percentage of the rescue analgesia needs of the groups.

While rescue analgesia was required for 49% of the children in the experimental group during the immediate postoperative period, only 13% of the children in the control group needed rescue analgesia, and this difference was found to be significant (*x*^2^ = 13.27, *p* < 0.05). However, no significant differences were observed between the two groups at the 1-hour postoperative and 2-hour postoperative time points (*p* > 0.05).  Figure 3 shows the percentages of the rescue analgesia needs of the groups.

The average recovery time was 17 ± 6.8 minutes for the experimental group and 13.2 ± 5.6 minutes for the control group, and this difference was found to be significant (*t* = −2.808, *p* = 0.006).

The average duration of the dental procedures was longer in the experimental group than in the control group, and the difference between them was statistically significant (*p* < 0.05) (see Table 2).

There was no significant relationship between the duration of the dental procedures and the recovery time (*p* < 0.05). However, there was a significant relationship between the duration of the dental procedures and the immediate postoperative pain scores (KW = 17.33, *p* = 0.002) and the rescue analgesic need (*t* = −3.720, *p* < 0.01).

## 4. Discussion

In this study, the postoperative pain scores, the need for rescue analgesia in the postoperative period, and the recovery times of children who were treated with deciduous molar pulpotomies were compared with those of children who underwent regular restorative dental treatment, all under GA. In addition, the effects of the duration of the procedures and postoperative pain on the recovery time were determined.

Various levels of postoperative pain were reported by 90% of the all patients. Higher postoperative pain scores (moderate to severe) were found in patients who underwent primary molar pulpotomies (49%) than in patients who underwent regular restorative treatment (13%), regardless of the number of procedures performed. Rescue analgesia was given according to the postoperative pain scores; however no local anesthesia was performed intraoperatively in any of the patients. This may be the cause of the high pain scores observed in pulpotomy cases, which is a painful dental procedure.

Although our study found that 90% of the groups experienced immediate postoperative pain, other researchers have reported levels of 86% and 70% of postoperative pain following dental extraction under GA [11, 12]. This difference was thought to have originated from the differences of the dental procedures used in these studies and also the timing of the evaluation. Our study included only patients who underwent pulpotomy and dental fillings in the experimental group and dental fillings only in the control group. However, the studies mentioned above included only patients who underwent tooth extraction. El-Batawi similarly searched for the postoperative pain in PDPs and they found the postoperative pain rate as 93.8% [10]. Moreover, the pain scores in our study were evaluated in the postoperative 1- and 2-hour periods and were seen to have displayed a drop in time and the difference between groups was noticed to continue. Any relation between the operation numbers and pain scores was not observed in intragroup evaluations.

Local anesthetic injections are frequently used during procedures performed under GA, especially tooth extraction, because the local anaesthetic assists with both bleeding control and postoperative pain relief [15]. However, the findings about the use of local anaesthesia for postoperative pain control are contradictory. Various authors such as Coulthard, McWilliams, and Townsend reported that local anaesthesia application did not affect postoperative pain scores [9, 15, 16]. However, Atan et al. [2] defended the view that local anaesthesia application in the perioperative period was effective in controlling pain. In addition, Watts and Kountakis stated that local anaesthesia applied during the perioperative period was effective in providing optimal haemodynamic parameters [17].

In our study, although IV analgesia was given in the intraoperative period, postoperative pain was reported in both groups but in children who underwent vital endodontic procedures higher pain scores were noted than control group. El Batawi [3] reported that the use of local anaesthesia together with IV analgesia decreased postoperative pain.

Prolonged recovery time in children causes increased postoperative anxiety and treatment costs because of the need for prolonged stays in hospital [18, 19]. Patients' recovery times varied according to the type of the dental procedures performed. Recovery times were longer in the experimental group, which underwent pulpotomies. This may have been because the postoperative pain was greater in the experimental group than in the control group [20].

There are several different methods for evaluating the pain of children in clinical studies such as self-reporting scales, behavioral assessment, and physiologic measurements [21]. In our study, the Wong-Baker FACES score was used to assess pain because previous studies have demonstrated that this pain assessment score is valid for children aged 3 to 7 years, and it is convenient to use [13, 22].

The ideal restorative material to be used after a primary molar pulpotomy is reported to be a stainless steel crown. However, stainless steel crowns do not meet today's rapidly increasing aesthetic demands. Other aesthetic crown coatings produced as alternatives to stainless steel crowns are not routinely used in clinical settings because of their high cost. For this reason, compomer resin was used to restore the pulpotomised teeth in the present study [23]. Previous studies have shown that if the edges of stainless steel crown remain to be long or the occlusion is poorly maintained, postoperative pain may occur [5, 6]. This situation may be confused with pulpotomy related postoperative pain. The possible confusion is avoided by not using stainless steel crown in our study.

## 5. Conclusions

This study is significant in that it indicates the need for effective postoperative pain control after vital endodontic treatment in comparison with the patients having restorative treatment only under GA. Postoperative pain control could be provided by giving additional postoperative analgesics following the intraoperative analgesics given to the patient. Postoperative pain results in a prolonged recovery period. Effective pain control is necessary to provide patients with a comfortable and fast recovery following vital pulp treatments performed under GA.

## Figures and Tables

**Table 1:** Components of Aldrete score scale.

Time	Score
*Activity*	
Able to move 4 extremities voluntarily or on command	2
Able to move 2 extremities voluntarily or on command	1
Unable to move extremities voluntarily or on command	0

*Respiration*	
Able to breathe deeply or cough freely	2
Dyspnea or limited breathing	1
Apneic	0

*Circulation*	
Blood pressure ±20% of preanesthetic level	2
Blood pressure ±20% to 49% of preanesthetic level	1
Blood pressure ±50% of preanesthetic level	0

*Consciousness*	
Fully awake	2
Arousable on calling	1
Not responding	0

*O*_*2*_ * saturation*	
Able to maintain O_2_ saturations >92% on room air	2
Needing O_2_ inhalation to maintain O_2_ saturations >90%	1
O_2_ saturation <90% even with O_2_ supplementation	0

Total score	10

**Table 2:** Comparison of the pain scores, rescue analgesic needs, duration of dental operation, recovery time of groups, and distribution of patient in terms of gender and mean age of children.

	*Experimental group N* (%)	*Control group N* (%)	*Chi-square/t*	*p value*
*Gender*				
Female	16(35.5)	22(48.8)	1.64/-	0.20
Male	29(64.5)	23(51.2)		

*Age *(mean ± SD)	4.9 ± 1.5	4.2 ± 1.8	-/1.748	0.08

*PIPS *(mean ± SD)	2.6 ± 1.7	1.3 ± 1.2	24.82/-	**<**0.01^**∗**^

*PPS (1 hour) *(mean ± SD)	1.6 ± 1.5	0.3 ± 0.7	-/4.99	**<**0.01^**∗**^

*PPS (2 hours) *(mean ± SD)	0.5 ± 0.6	0.08 ± 0.2	-/4.797	**<**0.01^**∗**^

*RAN*				
Present	22 (49)	6 (13)		
Absent	23 (51)	39 (87)	13.272/-	**<**0.01^**∗**^

*DDO (mean ± SD)*	56.6 ± 18.5	41.8 ± 14.7	-/4.204	**<**0.01^**∗**^

*RT (mean ± SD)*	17 ± 6.8	13.2 ± 5.6	-/2.808	0.01^**∗**^

**∗** = *p* < 0.05. RAN: rescue analgesia need, RT: recovery time, DDO: duration of dental operation, PIPS: postoperative immediate pain score, and PPS: postoperative pain score.

## References

[1] Çolak H., Dülgergil Ç. T., Dalli M., Hamidi M. M. (2013). Early childhood caries update: a review of causes, diagnoses, and treatments. *Journal of Natural Science, Biology and Medicine*.

[2] Atan S., Ashley P., Gilthorpe M. S., Scheer B., Mason C., Roberts G. (2004). Morbidity following dental treatment of children under intubation general anaesthesia in a day-stay unit. *International Journal of Paediatric Dentistry*.

[3] El Batawi H. Y. (2013). Lidocaine use for pain management during paediatric dental rehabilitation under general anaesthesia. *European Archives of Paediatric Dentistry*.

[4] Rodd H. D., Waterhouse P. J., Fuks A. B., Fayle S. A., Moffat M. A. (2006). Pulp therapy for primary molars. *International Journal of Paediatric Dentistry*.

[5] Ashkenazi M., Blumer S., Eli I. (2007). Post-operative pain and use of analgesic agents in children following intrasulcular anaesthesia and various operative procedures. *British Dental Journal*.

[6] Staman N. M., Townsend J. A., Hagan J. L. (2013). Observational study: discomfort following dental procedures for children. *Pediatric Dentistry*.

[7] Forrest J. B., Heitlinger E. L., Revell S. (1997). Ketorolac for postoperative pain management in children. *Drug Safety*.

[8] Leong K., Roberts G., Ashley P. (2007). Perioperative local anaesthetic in young paediatric patients undergoing extractions under outpatient 'short-case' general anaesthesia. a double-blind randomised controlled trial. *British Dental Journal*.

[9] Townsend J. A., Ganzberg S., Thikkurissy S. (2009). The effect of local anesthetic on quality of recovery characteristics following dental rehabilitation under general anesthesia in children. *Anesthesia progress*.

[10] El Batawi H. Y. (2015). Effect of intraoperative analgesia on children’s pain perception during recovery after painful dental procedures performed under general anaesthesia. *European Archives of Paediatric Dentistry*.

[11] Jensen B. (2012). Post-operative pain and pain management in children after dental extraction under general anaesthesia. *European Archives of Paediatric Dentistry*.

[12] O'Donnell A., Henderson M., Fearne J., O'Donnell D. (2007). Management of postoperative pain in children following extractions of primary teeth under general anaesthesia. a comparison of paracetamol, voltarol and no analgesia. *International Journal of Paediatric Dentistry*.

[13] Wong D. L., Baker C. M. (1988). Pain in children: comparison of assessment scales. *Pediatr Nurs*.

[14] World Health Organization (1997). *Oral Health Surveys: Basic Methods*.

[15] McWilliams P. A., Rutherford J. S. (2007). Assessment of early postoperative pain and haemorrhage in young children undergoing dental extractions under general anaesthesia. *International Journal of Paediatric Dentistry*.

[16] Coulthard P., Rolfe S., Mackie I. C., Gazal G., Morton M., Jackson-Leech D. (2006). Intraoperative local anaesthesia for paediatric postoperative oral surgery pain - a randomized controlled trial. *International Journal of Oral and Maxillofacial Surgery*.

[17] Watts T. L., Kountakis S. E. (2009). Intraoperative bupivacaine for reduction of post-tonsillectomy pain. a randomized, placebo-controlled, double-blind study of 26 patients. *Ear, Nose and Throat Journal*.

[18] Haddadi S., Marzban S., Karami M. S., Heidarzadeh A., Parvizi A., Nabi B. N. (2014). Comparing the duration of the analgesic effects of intravenous and rectal acetaminophen following tonsillectomy in children. *Anesthesiology and Pain Medicine*.

[19] Kocherov S., Hen Y., Jaworowski S. (2016). Medical clowns reduce pre-operative anxiety, post-operative pain and medical costs in children undergoing outpatient penile surgery: a randomised controlled trial. *Journal of Paediatrics and Child Health*.

[20] Boroumand P., Zamani M. M., Saeedi M., Rouhbakhshfar O., Motlagh S. R. H., Moghaddam F. A. (2013). Post tonsillectomy pain: Can honey reduce the analgesic requirements?. *Anesthesiology and Pain Medicine*.

[21] McGrath P. J., Cunningham S. J., Goodman J. T., Unruh A. (1986). The clinical measurement of pain in children. *The Clinical Journal of Pain*.

[22] Wilson M. E., Helgadóttir H. L. (2006). Patterns of Pain and Analgesic Use in 3- to 7-Year-Old Children After Tonsillectomy. *Pain Management Nursing*.

[23] Fernandes A. P., Lourenço Neto N., Teixeira Marques N. C. (2015). Clinical and radiographic outcomes of the use of Low-Level Laser Therapy in vital pulp of primary teeth. *International Journal of Paediatric Dentistry*.

